# Controlled ovulation of the dominant follicle using progestin in minimal stimulation in poor responders

**DOI:** 10.1186/s12958-017-0291-0

**Published:** 2017-09-05

**Authors:** Qiuju Chen, Yun Wang, Lihua Sun, Shaozhen Zhang, Weiran Chai, Qingqing Hong, Hui Long, Li Wang, Qifeng Lyu, Yanping Kuang

**Affiliations:** Department of Assisted Reproduction, Shanghai Ninth People’s Hospital, Shanghai Jiaotong University School of Medicine, Zhizaoju road no 639, Shanghai, People’s Republic of China

**Keywords:** Poor responder, Natural cycle, Progestin, Premature ovulation

## Abstract

**Background:**

The use of progestin (P) during ovarian stimulation is effective in blocking the luteinizing hormone (LH) surge in women with normal ovarian reserve, however, its effects have not been determined in poor responders. This study aimed to explore the follicular dynamics in P-primed minimal stimulation in poor responders.

**Methods:**

A total of 204 infertile women with diminished ovarian reserve were allocated into the medroxyprogesterone acetate (MPA) group or the natural-cycle control group in an alternating order. MPA (10 mg) was administered daily beginning from the early follicular phase and a low dose of hMG was added in the late follicular phase if the serum FSH level was lower than 8.0mIU/ml. When a dominant follicle reached maturity, triptorelin 100 μg and hCG 1000 IU were used for trigger, and oocytes were retrieved 34-36 h later.All viable embryos were cryopreserved for subsequent frozen embryo transfer. Natural cycle IVF was used as controls.

**Results:**

Compared with the natural cycle group, the MPA group exhibited a larger pre-ovulatory follicle (18.7 ± 1.8 mm vs 17.2 ± 2.2 mm), a longer follicular phase (13.6 ± 3.6 days vs 12.3 ± 3.2 days), and higher peak oestradiol values (403.88 ± 167.16 vs 265.26 ± 122.16 pg/ml), while maintaining lower LH values (*P* < 0.05). The incidences of spontaneous LH surge and premature ovulation decreased significantly (1.0% vs 50%; 2% vs. 10.8%, respectively; *P* < 0.05). A greater number of oocytes and viable embryos were harvested from the MPA group than from the natural cycle group (*P* < 0.05). Moreover,the clinical pregnancy rate was slightly higher in the MPA group than in the natural cycle controls, but the difference was not significant (11.8% vs 5.9%, *P* > 0.05).

**Conclusion:**

This study supported the hypothesis that P-primed minimal stimulation achieved ovulation control of the dominant follicle and did not adversely affect the quality of oocytes in poor responders. Therefore, P-priming is a promising approach to overcome premature ovulation in minimal stimulation for poor responders.

**Trial registration:**

ChiCTR-OCH-14004176. Registered on January 8, 2014.

## Background

Improvements in cryopreservation techniques for assisted reproductive technology (ART) have allowed reproductive physicians to consider a new strategy of using progestin (P) as an oral alternative to GnRH analogues for improving in vitro fertilization (IVF) practices [1–7]. P can be used in two ways: whether it be endogenously (as in the luteal-phase stimulation) or exogenously (as in the use of P in the follicular phase) [1, 2]. P-primed ovarian stimulation (PPOS) protocols have been confirmed to effectively block the rise of luteinizing hormone (LH) and demonstrate a comparable pregnancy outcome to that of classical protocols for infertile women with normal ovarian reserve and polycystic ovarian syndrome [1, 2, 4–7]. However, the potential usefulness of PPOS protocols in clinical practice needs to be determined, for example, there is a lack of relevant data regarding its efficacy in poor responders.

Several reports confirm that poor responders cannot benefit from increasing gonadotropin doses; thus, natural cycle IVF or minimal stimulation is a patient-friendly option, especially for patients with low antral follicle counts [8–12]. Natural cycle IVF faces the problems of untimely premature ovulation and individualized schedules for oocyte retrieval [13]. Although a modified natural cycle with a GnRH antagonist at the mid-late follicular phase has lowered the occurrenceof premature ovulation, this problem has still not been completely resolved [8, 10, 12]. Oral P has been demonstrated to effectively block LH rise and premature ovulation in controlled ovarian stimulation [2]. Therefore, we explored the role of P in blocking premature ovulation in minimal stimulation for poor responders.

The goal of P-primed minimal stimulation is to develop a single dominant follicle in the P-primed status, and a low dose of hMG is administered at the late follicular phase to avoid possible suppression by P. To explore follicular phase dynamic changes in the P-primed status, natural cycle IVF was used as a blank control to compare follicle growth dynamics with or without P-primed minimal stimulation.Therefore, a prospective controlled study was performed to investigate the follicular and endocrinological characteristics of P-primed minimal stimulation in poor responders.

## Methods

### Subjects

A prospective cohort study was conducted at the Department of Assisted Reproduction of the Ninth People’s Hospital of Shanghai Jiaotong University School of Medicine. Women undergoing IVF/intracytoplasmic sperm injection (ICSI) regimens for the treatment of infertility were recruited between January 2014 and December 2014. The study protocol was approved by the Ethics Committee (Institutional Review Board) of the Ninth People’s Hospital of Shanghai and registered with the Chinese Clinical Trial Registry (ChiCTR-OCH-14004176). The trial was conducted according to the Declaration of Helsinki for medical research. All participants provided informed consent after undergoing counselling for infertility treatments and routine IVF procedures.

Patients planning to undergo IVF/ICSI treatments were screened for eligibility by transvaginal ultrasound and serum hormone testing on menstrual cycle day 3. Participants met the following criteria: 1) aged 25–45 years; 2) spontaneous menstrual cycle (21–35 days in duration); and 3) bilateral antral follicle counts (AFC) <5 on menstrual cycle day 3 and a basal serum follicle-stimulating hormone (FSH) concentration between 10 and 30 mIU/ml. We excluded cases with higher basal oestradiol (E_2_) levels (E_2_ > 70 pg/ml) or ovarian functional cysts on menstrual cycle day 3 because the higher basal E_2_ values would potentially interfere with the ability of P to suppress pituitary function [2].

Patients were recruited consecutively and assigned to one of two groups, namely, the medroxyprogesterone acetate (MPA) group or the natural cycle group, in an alternating manner. Odd-number-assigned patients were allocated to the MPA group, and even-number-assigned patients were allocated to the natural cycle group. Each woman completed only one cycle in this trial.

### Sample size estimate

For the power calculation, previous studies reported that 17% of oocyte retrievals were cancelled due to a premature LH surge or ovulation in natural cycle IVF patients [13]. Because there were no relevant data regarding the efficacy of MPA in poor responders, we hypothesized that the administration of MPA would decrease the incidence of premature LH surges and ovulation to 8%; therefore,the superiority margin was set at 8%. A sample size of 97 in each group would yield 90.0% power to establish superiorityat the 0.01 level of significance. Assuming a drop-out rate of approximately 5%, the number of participants needed was 102 subjects in each group in this trial.

### Protocol

#### Natural cycle IVF

In the natural cycle group, oocyte pick-up (OPU) was performed within a pure natural cycle that excluded any hormonal stimulation except for GnRH agonist (GnRHa) administration for ovulation induction. Ultrasound monitoring began on cycle day 7 until the dominant follicle presented. When the follicle reached a diameter of 13 mm or greater and the E_2_ exceeded 150 pg/ml, patients were monitored every day or every other day. When the dominant follicle reached 18 mm in diameter, in the absence of a LH rise, the final stage of oocyte maturation was induced around midnight with 100 μgof triptorelin (Decapeptyl, Ferring GmbH, Germany). A nonsteroidal anti-inflammatory drug, ibuprofen (0.6 g), was used on the trigger day and the following day.Transvaginal ultrasound-guided oocyte retrieval was scheduled 32-36 h later. For cases with a mature follicle and the occurrence of a spontaneous LH surge (LH >20 mIU/ml), GnRHa was not administered, and only ibuprofen was used. Oocyte retrieval was arranged 18-30 h later, according to the presumed stage of the spontaneous LH surge on the scheduled day [14].

### MPA protocol

MPA(10 mg) was administered daily from menstrual cycle day 3 onwards. After 5 days, transvaginal ultrasonography and serum hormone measurements were performed every 2–4 days.When the follicle began to grow, the E_2_ level increased, and FSH significantly decreased to ≤8.0 mIU/ml, a low dose of hMG(75-150 IU/d) was administered to promote late follicular development. When the dominant follicle reached a diameter of 18 mm, the final stage of oocyte maturation was stimulated with triptorelin (100 μg) and hCG (1000 IU). Oocyte retrieval was performed 34-36 h later. The co-trigger method was based on our previous studies [2].

### In vitro fertilization and embryo culture

Before oocyte retrieval, the presence of a dominant follicle was confirmed by transvaginal ultrasound. If the dominant follicle disappeared, it was assumed that premature ovulation had occurred before the scheduled time. Oocyte retrieval was performed without sedation or local anaesthesia with a double-lumen aspiration needle. The follicle was flushed three times at most if no cumulus cell oocyte complexes (COC) were present. All follicles >10 mm in diameter were retrieved. If an oocyte was obtained, standard insemination or ICSI was performed within 6 h of retrieval. Embryos were examined for the number and regularity of the blastomeres and the degree of embryonic fragmentation. All top-quality embryos (including grade I and grade II 8-cell blastomere embryos) were frozen by vitrification on the third day following oocyte retrieval. The non-top-quality embryos were placed in extended culture, and only blastocysts with good morphology were frozen on day 5 or day 6. The cryopreservation procedure has been described previously [2]. Hormone replacement treatment was recommended for endometrial preparation. Briefly, ethiny estrogen (25 μg tid) was administered for 14 days, and the treatment was then shifted to oral progesterone (4 femoston yellow tablets daily, including 8 mg oestradiol and 40 mg dydrogesterone) and soft vaginal progesterone capsules (200 mg bid). Day-3 embryo transfer was arranged three days later. Blastocyst transfers were performed on the fifth day. Once pregnancy was achieved, exogenous oestrogen and progesterone supplements were continued until 10 weeks of gestation.

### Laboratory analysis

Serum FSH, LH, E_2_ and progesterone levels were collected on cycle day 3, cycle days 8–10, the trigger day and the following day (approximately 10 h after the trigger). In the natural cycle group, multiple hormone measurements were obtained during the late follicular phase. Hormone levels were measured by chemiluminescence (Abbott Biologicals B.V., The Netherlands). The lower limits of sensitivity were as follows: FSH = 0.06 mIU/ml, LH = 0.09 mIU/ml, E_2_ = 10 pg/ml and *P* = 0.1 ng/ml.

### Statistical analysis

The primary measurement in this study was the incidence of spontaneous LH surge and premature ovulation.The secondary measurement included the dynamic characteristics of the steroid hormone profiles, the number of retrieved oocytes and the number of viable embryos. Spontaneous LH surge was defined as LH > 20 mIU/ml during minimal stimulation. Premature LH surge was defined as LH >20 mIU/ml when the dominant follicle was <15 mm in diameter. Premature ovulation was defined as a follicle rupture before the scheduled time.

Efficacy analysis was based on the intention-to-treat (ITT) population. Analyses of the primary and secondary endpoints were carried out for each initiating cycle. Student’s *t*-test and the Man-Whitney *U*-test were used for normal and non-normal distributions, respectively.The Kruskal-Wallis test was used for comparison of hormone profiles, and the chi-square test was used for binary variable comparisons. A *P*-value <0.05 indicated statistical significance. All data were analysed using the Statistical Package for the Social Sciences for Windows (SPSS, Version 16.0, SPSS Inc., Chicago, IL, USA).

## Results

### Basic patient characteristics

A total of 204 women were enrolled and allocated to either the natural cycle or MPA group according to the study protocol. In the MPA group, the average age was 37.3 ± 4.7 years, the basal FSH was 13.3 ± 5.8 mIU/ml, and the AFC was 2.2 ± 1.4. A total of 77.4% (79/102) of the women had previously undergone unsuccessful IVF/ICSI attempts, including 34 women who had failed at least 3 times.There were no statistically significant differences in the basic characteristics between the MPA and control groups (Table 1).

**Table 1:** Baseline characteristics of poor responders undergoing IVF/ICSI treatment

	Natural cycle group (*n* = 102)	MPA group (n = 102)	*P* value
Age (years)	37.8 ± 4.7	37.3 ± 4.7	0.440
Duration of infertility (years)	5.0 ± 4.2	4.8 ± 3.2	0.711
BMI (kg/m^2^)	21.9 ± 2.5	21.2 ± 3.1	0.089
Basal FSH (mIU/ml)	11.9 ± 6.5	13.3 ± 5.8	0.100
Basal E_2_ (pg/ml)	39.8 ± 25.4	33.7 ± 21.6	0.076
Antral follicle counts	2.5 ± 2.0	2.2 ± 1.4	0.165
Type of infertility (%)			0.068
Primary	40 (39.2)	53 (52.0)	
Secondary	62 (60.8)	49 (48.0)	
Indications (%)			0.145
Tubal	46 (45.1)	36 (35.3)	
Male	11 (10.8)	16 (15.7)	
Endometrosis	17 (16.7)	22 (21.6)	
Ovulation dysfunction	12 (11.8)	5 (4.9)	
Unknown	7 (6.9)	6 (5.9)	
Combined	9 (8.8)	17 (16.7)	
Previous IVF failure (%)			0.152
0	16 (15.7)	23 (22.5)	
1–2	39 (38.2)	45 (44.1)	
> 3	47 (46.1)	34 (33.3)	

Values are expressed by means and standard deviations (SD) or n (%)

### Dynamic characteristics of thehormone concentrations

**Fig. 1:**
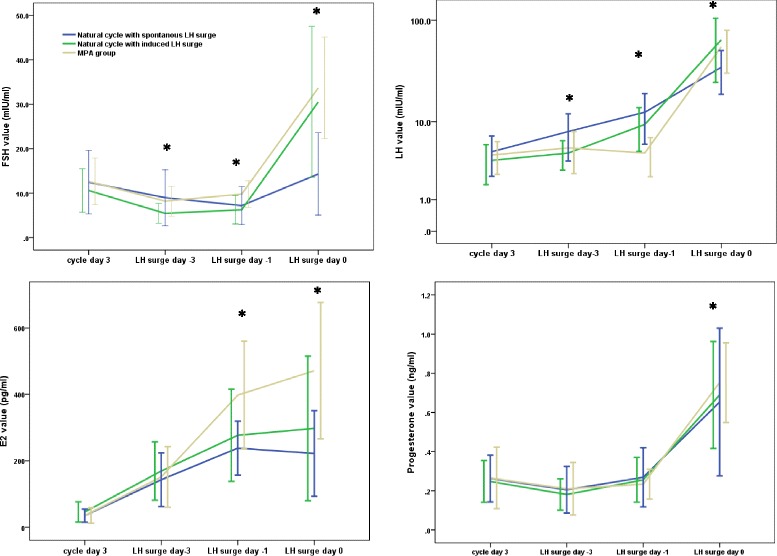
Hormone profiles during the natural cycle and P-primed minimal stimulation. Cases with a spontaneous LH surge in the natural cycle group are marked as blue lines, and cases with an induced surgeare marked as green lines.Cases in the MPA group are marked with yellow lines. The spontaneous or induced LH surge day is recorded as day 0

In the natural cycle control group, the growth of one or two dominant follicles was accompanied by a gradual increase of E_2_ levels in 101 cases, except for one case in which there was no follicle growth. Serum FSH values decreased slightly during the follicular phase, and LH values demonstrated a gradual increasing trend; 50% of all cases (51/102) presented a spontaneous LH surge (>20 mIU/ml), with an average LH peak value of 35.1 ± 16.0 mIU/ml, and the patients underwent oocyte retrieval after 18-30 h based on the presumed stage of ovulation.The remaining 50 cases in the natural cycle group did not exhibit spontaneous LH surges, their mean LH level on the trigger day was 9.27 ± 4.52 mIU/ml, which was close to the onset of the LH surge. These patients were triggered and oocyte retrieval was completed 32-36 h later. Therefore, based on the diagrams of the hormone profiles, the natural cycle group was divided into two subgroups: a spontaneous LH surge and an induced LH surge (Fig. 1). In Fig. 1, the LH surge day is denoted day 0, and the trigger day is denoted LH-1. In the subgroup of the natural cycle women with a spontaneous LH surge, E_2_ values reached a peak one day before the LH surge. In the subgroup of the natural cycle women with an induced LH surge, the E_2_ values continued to increase after the trigger.

In the MPA group, the dominant follicle continued to grow in 101 cases, except for one case with no developing follicle. Serum FSH levels during the later follicular phases (LH surge day-3, day −1 and day 0) were higher than those in the natural cycle group (*P* < 0.05). The mean LH value on the trigger day was 4.75 ± 2.36 mIU/ml and the median value was 4.11mIU/ml. The incidences of spontaneous LH surges were lower in the MPA group than in the natural control group (1.0% vs 50.0%, *P* < 0.05). Serum LH values in the later follicular phases were significantly lower in the MPA group than in the natural control group (*P* < 0.05). The observed trend in LH level changes during the follicular phase was either stable or slightly increasing, and the ratio of the LH value on the trigger day to the basal LH level was significantly lower in the MPA group than in the control group (*P* < 0.01).

E_2_ levels demonstrated an increasing trend in the MPA group. Peak E_2_ values in the MPA group were significantly higher than the values in the natural cycle group (403.88 ± 167.16 pg/ml vs 265.26 ± 122.16 pg/ml, *P* < 0.05). Progesterone levels were consistently in the lower range during the follicular phase in both groups; however, progesterone values in the MPA group were slightly higher after the trigger (*P* < 0.05).

### Follicular dynamics during P-primed minimal stimulation

Compared with the natural cycle group, the duration of the follicular phase in the MPA group was one day longer (13.6 ± 3.6 days vs. 12.3 ± 3.2 days, *P* < 0.05).The numbers of pre-ovulatory follicles were comparable (1.2 ± 0.5 vs. 1.0 ± 0.3, *P* > 0.05), and the average diameter of the pre-ovulatory follicle on the trigger day was larger (18.7 ± 1.8 mm vs. 17.2 ± 2.2 mm, *P* < 0.05). Additional evidence of controlled ovulation was the interval between the trigger and oocyte retrieval in the MPA group, which remained steady (35.4 ± 0.6 h). In the natural cycle group, the interval depended on the hormone profile and expected ovulation time; thus, the oocyte retrieval timing varied from 18 to 36 h after spontaneous or induced LH surge. Although multiple treatments were administered in the natural cycle control group (using nonsteroidal anti-inflammatory drugs and advanced oocyte retrieval), the incidences of premature ovulation were significantly higher in the natural cycle group than in the MPA group (10.8% vs 2.0%, *P* < 0.05).

### Association between serum LH level and pre-ovulatory follicle diameter

**Fig. 2:**
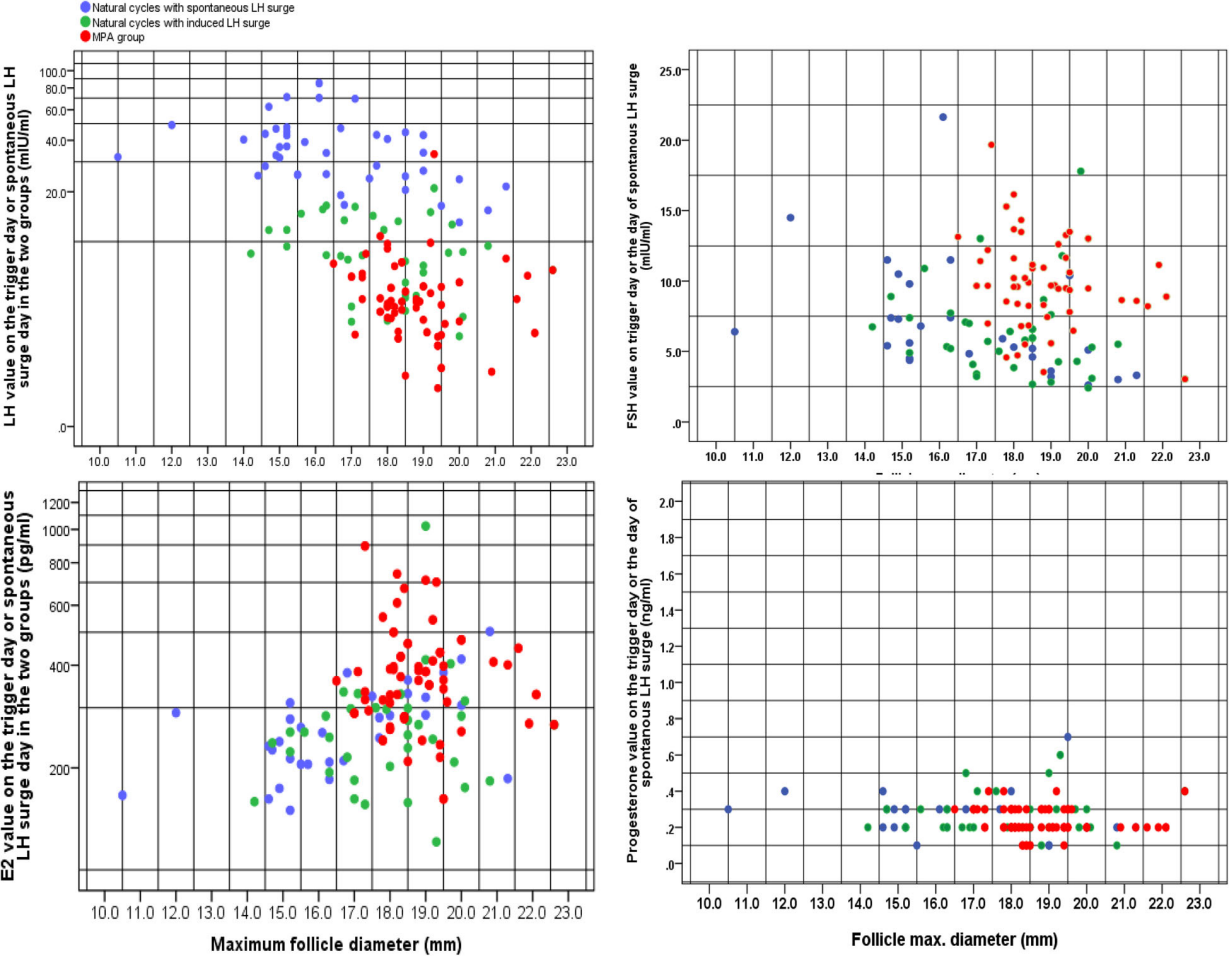
Scatterplot of maximum follicle diameter and serum hormones (FSH, LH, E_2_ and progesterone) on the scheduled day. In the natural cycle group, cases with a spontaneous LH surge are marked as blue spots, and cases with a GnRHa trigger are marked as green spots. Cases in the MPA group are marked as red spots. The vertical axis represents the hormone levels on the scheduled day (log scale). The horizontal axis represents the maximum diameter of the follicle

Correlations between the maximum diameter of the follicle and serum hormones (FSH, LH, E_2_ and progesterone values) on the trigger day or spontaneous-LH-surge day in the two groups are shown in Fig. 2. Briefly, 8.8% (9/102) of the cases in the natural cycle group had smaller dominant follicles with premature LH surges (the diameter of the pre-ovulatory follicles < 15 mm), indicating that poor responders tend to ovulate with a smaller mean follicle diameter in the natural cycle. In contrast, no events of small pre-ovulatory follicles and premature LH surges occurred in the MPA group (*P* < 0.05), which is further evidence of well-controlled ovulation of the dominant follicle using P-primed minimal stimulation.

### IVF performance

Table 2 displays the cycle characteristics of the two regimens. A total of 77 oocytes were retrieved from 73 women in the natural cycle group, whereas 103 oocytes were retrieved from 87 women in the MPA group. The mean MPA duration was 9.2 ± 2.3 days, and the hMG duration was 2.9 ± 2.0 days.The mean hMG dose administered to the MPA group was 357.49 ± 272.4 IU. The numbers of oocytes retrieved, metaphase II (MII) oocytes, fertilization and viable embryos were significantly higher in the MPA group than in the natural cycle group. However, no between-group differences were observed in the proportion of mature oocytes (88.9% vs. 84.4%), fertilization rate (76.2% vs. 69.0%) and cleavage rate (96.1% vs. 98.0%) (*P* > 0.05).The number of viable embryos was significantly higher in the MPA group than in the control group (*P* < 0.05).The proportion of cycles with at least one viable embryo in the MPA group was higher than that in the natural cycle group, but this difference was not significant (50.0% vs. 38.3%, *P* > 0.05).

**Table 2:** IVF Outcomes of natural cycle and P-primed minimal stimulation in poor responders

	Natural cycle group (n = 102)	MPA group (n = 102)	P value
No of >10 mm follicles on trigger day	1.18 (1.08, 1.27)	1.37 (1.24, 1.50)	0.012
No of >14 mm follicles on trigger day	1.03 (0.96, 1.09)	1.16 (1.07, 1.25)	0.025
No of oocytes retrieved	0.76 (0.65,0.86)	1.09 (0.93,1.18)	<0.001
No of immature oocytes	0.05 (0, 0.1)	0.02 (−0.01,0.05)	0.307
No of MII oocytes	0.64 (0.53,0.74)	0.94 (0.81,1.07)	<0.001
No of abnormal oocytes	0.06 (0,0.08)	0.09 (0.03, 0.15)	0.155
No of fertilized oocytes	0.48 (0.38, 0.58)	0.76 (0.63,0.88)	0.001
No of viable embryos	0.89 (0.79, 0.98)	1.10 (1.10, 1.20)	0.003
Clinical pregnancy per patient (%)	5.88% (6/102)	11.77% (12/102)	0.139
Implantation rate (%)	15.38% (6/39)	21.43% (12 /56)	0.460
Miscarriage rate (%)	33.33% (2/6)	8.33% (1/12)	0.245
Ectopic pregnancy (%)	0 (0/6)	8.33% (1/12)	0.667
Live birth rate per patient (%)	3.92% (4/102)	8.33% (10/102)	0.097

Values are expressed by means (95% CI) or % (n);

Forty-two viable embryos in the natural cycle group and 56 viable embryos in the MPA group were cryopreserved for frozen embryo transfer (FET). The MPA group demonstrated a slightly higher clinical pregnancy rate than the natural cycle group, but this difference was not significant due to the small sample size (11.8% (12/102) vs. 5.9% (6/102), *P* < 0.05). The live birth rate was 8.3% (10/102) in the MPA group and 3.9% (4/102) in the natural control group (*P* > 0.05). The implantation rate was comparable in the two groups, indicating that the administration of P did not adversely affect the embryo developmental potential in the poor responders (21.4% vs. 15.4%, *P* > 0.05).

## Discussion

In this trial, we prospectively compared the follicular phase dynamics of P-primed minimal stimulation and natural cycle IVF in poor responders.The results indicated that ovulation of the dominant follicle in P-primed minimal stimulation was well-controlled and facilitated the IVF programme. IVF results demonstrated that the MPA group produced more oocytes and embryos than the control group, and the comparable pregnancy outcome indicated that P did not adversely affect oocyte quality. Therefore, P-priming is a promising approach toward overcoming premature ovulation during minimal stimulation for poor responders.

In this trial, we included participants with diminished ovarian reserve (with high FSH values and AFC < 5) who still had regular menstrual cycles.The criteria for this trial were under the widely-used Bologna criteria, and a regular cycle was listed as one of the inclusion criteria for it was one of the prerequisites for performing natural cycle IVF. These poor responders had small quantities of primordial follicle pools and FSH-sensitive follicles, wherein the follicles biologically “matured” quickly and were prone to experience premature luteinization [15, 16]. The reproductive hormone profiles demonstrated higher FSH levels in the early follicular phase, exaggerated amplitudes, protracted LH durations, declined E_2_ and progesterone levels and shared similarities with profiles described during menopausal transition [17]. These poor responders provide a good model for investigating single follicle development and ovulation using P treatment.

Natural cycle IVF is aimed at developing a naturally mature follicle, and the timing of oocyte retrieval is dependent on the spontaneous LH surge. A spontaneous LH surge cannot be clearly predicted; therefore, follicle monitoring was frequently performed in the late follicular phase, and 50% of the cases presented with a spontaneous LH surge. Oocyte retrieval was arranged according to the presumed stage of spontaneous ovulation [14].The remaining patients did not present spontaneous LH surges until the follicle reached a diameter of 18 mm, we then triggered and arranged oocyte retrieval, as is routinely performed. Thus, half of the cases demonstrated a spontaneous LH surge in the natural control group. The process of waiting for follicles to mature gave us a good opportunity to distinguish the changes incurred by MPA.

In this trial, ovulation of the dominant follicle in the MPA group was controlled, as indicated by the following evidence: a single large follicle developed, spontaneous LH surge was prevented (1.0%), and ovulation and luteinization rarely occurred unless a GnRHa/HCG bolus was administered. Moreover, P-priming prolonged the follicular phase by one more day, and the diameter of the pre-ovulatory follicle was larger than that of the natural cycle patients.These data indicated that MPA treatment significantly suppressed follicular rupture, which undoubtedly postponed hCG administration with less concern for the development of a premature LH surge. This treatment enabled most of the oocytes to achieve greater maturity before aspiration. Thus, P-primed minimal stimulation provides a wide window for oocyte retrieval.

The normal mechanism of follicle rupture may be disturbed by continuous exogenous administration of P [18–22], which may be due to at least two factors. First, P-priming during the follicular phase prevents the occurrence of the GnRH surge even when the quantity of oestradiol administration is well above that needed for surge induction [19]. P-priming slows the LH pulse frequency, augments the pulse amplitude and reduces the mean plasma LH levels compared with those in untreated women [20]. In this trial, the decreased LH values and low incidences of spontaneous LH surges in the MPA group are signs of the hypophyseal suppression that is induced by exogenous P or the combined action of oestradiol and P. Second, the continuous administration of P may directly interfere with a follicle’s spontaneous rupture by modulating the progesterone receptor (PR). In PR-null mice, the follicles mature to the pre-ovulatory stage in response to exogenous gonadotropins; however, in the absence of PR signalling, they fail to rupture and release oocytes [21].Ulipristal acetate, which is a progesterone receptor modulator, blocks ovulation by inhibiting PR-dependent pathways that are intrinsic to the ovary when administered within a critical time window following the LH surge (<6 h) [22].

Most studies demonstrate that follicular development continues during treatment with hormonal contraceptives because the P contained in most combined oral contraceptives has a low influence on FSH secretion [23]. In this trial, the patients had higher basal FSH levels and follicular-phase FSH values were maintained near the physiological range (5–10 mIU/ml) [24], most cases demonstrated that follicular growth continued after the daily administration of 10 mg MPA. The possibility of P-mediated inhibition of dominant follicle development has been reported in animal models [25], therefore, hMG was administered during the late follicular phase to avoid regression of the dominant follicle.

In this trial, the oocyte/embryo results reflected the efficacy of the different treatment strategy.Oocyte retrieval in natural cycle is individualized based on the occurrence of a spontaneous LH surge; therefore, frequent monitoring and a 7-day-per-week working schedule are unavoidable for natural cycle IVF. A large studyof 1048 patients who underwent modified natural-cycle IVF demonstrated a 66% oocyte retrieval rate per scheduled retrieval and confirmed that the use of GnRH antagonists did not substantially improve the outcome [9].Our trial demonstrated an efficacy of 71.57% for the retrieval rate and 38.24% for the embryo transfer rate in natural cycle IVF patients, which is in accordance with previous reports [9–12]; the MPA group demonstrated superior oocyte and embryo outcomes. Notably, the higher number of retrieved oocytes and embryos may be associated with the administration of hMG during the late follicular phase of the MPA treatment. However, the rate of successfully retrieved, mature oocytes and cleaved embryos was comparable between the two groups, and the embryonic developmental potential was approved by the slightly better or similar clinical pregnancy rate and live birth rate.These data suggest that P-priming does not adversely affect oocyte quality. Another retrospective trial of 993 cycles of P-primed mild stimulation in our clinic demonstrated no adverse effects of P on the embryo developmental potential in poor responders (unpublished data).

One limitation of this trial is its quasi-randomized nature. Some potential confounding factors could not be excluded due to the alternating group assignments. The sample size was designed to have sufficient power to distinguish the difference in the presence of LH surge; however, it was underpowered for comparisons of pregnancy outcomes.Therefore, our trial focused on a detailed observation of follicular dynamics and endocrinological profiles. Thus, the results of the embryos and pregnancy outcomes should be interpreted with caution.

## Conclusions

In poor responders undergoing P-priming minimal stimulation, the follicle continuously grows and appears more robust, and spontaneous LH surges and premature ovulation are inhibited. Oocyte quality is not adversely affected by continuous administration of P. This treatment provides a novel insight into the prevention of premature ovulation and improvement in the IVF programme for poor responders, although questions regarding possible effects on the embryo developmental potential remain to be investigated. A well-designed randomized controlled trial should be performed to compare the efficacy of P and GnRH antagonists in preventing premature ovulation in poor responders.

## Abbreviations

AFC: Antral follicle count
AMH: Anti-Mullerian hormone.
FET: Frozen embryo transfer
FSH: Follicle-stimulating hormone
ICSI: Intracytoplasmic sperm injection
IVF: In vitro fertilization
LH: Luteinizing hormone
MPA: Medroxyprogesterone acetate
P: Progestin
